# A polarization-based image restoration method for both haze and underwater scattering environment

**DOI:** 10.1038/s41598-022-05852-1

**Published:** 2022-02-03

**Authors:** Zhenming Dong, Daifu Zheng, Yantang Huang, Zhiping Zeng, Canhua Xu, Tingdi Liao

**Affiliations:** 1 Fujian Science & Technology Innovation Laboratory for Optoelectronic Information of China, Fuzhou, 350108 Fujian People’s Republic of China; 2 College of Physics and Information Engineering, Fuzhou University, Fuzhou, 350108 Fujian People’s Republic of China; 3 Research Center for Photonics Technology, Quanzhou Normal University, Quanzhou, 362000 Fujian People’s Republic of China; 4 Fujian Provincial Collaborative Innovation Center for Ultra-Precision Optical Engineering and Applications, Quanzhou, 362000 Fujian People’s Republic of China

**Keywords:** Applied optics, Optical techniques, Imaging and sensing

## Abstract

Existing polarization-based defogging algorithms rely on the polarization degree or polarization angle and are not effective enough in scenes with little polarized light. In this article, a method of image restoration for both haze and underwater scattering environment is proposed. It bases on the general assumption that gray variance and average gradient of a clear image are larger than those of an image in a scattering medium. Firstly, based on the assumption, polarimetric images with the maximum variance (*I*_*best*_) and minimum variance (*I*_*worst*_) are calculated from the captured four polarization images. Secondly, the transmittance is estimated and used to remove the scattering light from background medium of *I*_*best*_ and *I*_*worst*_. Thirdly, two images are fused to form a clear image and the color is also restored. Experimental results show that the proposed method obtains clear restored images both in haze and underwater scattering media. Because it does not rely on the polarization degree or polarization angle, it is more universal and suitable for scenes with little polarized light.

## Introduction

In traffic monitoring, remote sensing and ocean exploration, because of the scattering medium, such as fog, haze, turbid water and so on, the visibility and the contrast of images are reduced and the details of images are blurred^1–3^. Usually there are two types of imaging methods to enhance the image clearness^1^. One is built on image enhancement algorithm, such as histogram equalization^4^ and Retinex algorithm^5^. The other is image restoration methods, which obtain unscattered object light based on specific physical models or priori hypotheses, such as dark channel prior method, Schechner's polarization defogging method, and Tan's single image defogging method. The image enhancement methods are straight forward and very effective to improve the contrast of blurred images. However, because these methods do not take into consideration of the image degeneration, they usually induce more image distortion and obtain fewer recovery details than image restoration methods. In addition, defogging methods based on using deep learning technologies become prevail in recent years^6,7^.

In this article, we present a defogging algorithm in the scope of image restoration method. Plenty of researches on image restoration techniques through scattering mediums have been conducted over the past decades. Classic methods are listed as following. In 2001, Schechner et al.^8–10^ proposed an image defogging method based on polarization difference. They assumed that the atmospheric light is partially polarized and the object light is unpolarized. The difference between two polarization images (“*I*_*max*_” and “*I*_*min*_”) is regarded as atmospheric polarized light. And an Atmospheric Propagation Model is adopted to restore the image. In the next few years this method was extended to be used in the field of underwater imaging experiments^11^. In 2008, Tan^12^ proposed a single image defogging method based on two assumptions of that clear images have larger contrast than foggy images and the airlight tends to be smooth in foggy weather. In the same year, Fattal^13^ formulated a refined image formation model. They introduced the new variable “surface shading” and assumed that “surface shading” and transmission functions are locally statistically uncorrelated. Then the defogging is conducted with the transmission functions calculations. The well-known image dehazing method using dark channel prior^14^ was proposed by K. He et al. in 2009. An End-to-End System for Single Image Haze Removal using deep learning was presented by Cai et al. in 2016^6^. In very recent years, Zhu et al.^15,16^ proposed a novel fast single image dehazing algorithm based on artificial multiexposure image fusion and an image dehazing method by an artificial image fusion method based on adaptive structure decomposition. Vazquez-Corral et al.^17^ proposed a method of physical-based optimization for non-physical image dehazing methods. In 2021, by using dual self-attention boost residual octave convolution, Zhu et al.^18,19^ developed a defogging method suitable for remote sensing imaging.

In general, benefit from more information obtained from multiple different polarization images, restoration method based on polarization imaging has more advantages and better details than single image dehazing methods. This technology had been intensively researched in past decades in the aspects of visibility enhancement^20^, active imaging system^21,22^, underwater target detection^23–25^, real-time measurement^26^, long-range polarization imaging^27^, utility of the polarization angle^28–30^. Moreover, Shao et al.^31^ proposed a hazy image restoration method based on atmospheric light polarization tomography. Liu et al.^32^ used Wavelet Transform to stratify images and removed haze of images. Fang et al.^33^ proposed an image dehazing method using polarization effects of objects and airlight. In 2021, Liang et al.^34^ proposed a low-pass filtering based polarimetric dehazing method for dense haze removal. So far plenty of methods have been used to restore the image effectively in the specific scene. The restoration effect depends on the accurate estimation of polarization degrees or polarization angles. However, it is difficult to calculate the polarization parameters exactly in a strong scattering scene with very weak polarized light. Large polarization parameters estimation error results in unsuccessful image restorations in these scenes. In comparison, calculation of gray variance is more accurate and practical in the various conditions. Based on the general assumption that gray variance and average gradient of a clear image are larger than those of an image through scattering media, a universal image restoration method is proposed in this article. Firstly, Stokes parameters are used to find polarimetric images with the maximum variance (*I*_*best*_) and minimum variance (*I*_*worst*_). Secondly, the transmittance can be estimated and be used to remove the scattering light from background medium of *I*_*best*_ and *I*_*worst*_. Thirdly, two images are fused to form a clear image and the color is also restored. Experimental results show that the proposed method is applicable both in atmospheric and underwater scattering media. Moreover, it is still very effective in scenes with little polarized light.

## Technical background

### Stokes parameters

The polarization properties of light can be described by the Stokes parameters. The Stokes parameters of light are denoted as [*S*_0_, *S*_1_, *S*_2_, *S*_3_]^T^, where *S*_0_ is the total light intensity, *S*_1_ is the light intensity difference between 0° and 90° polarization direction, *S*_2_ is the light intensity difference between 45° and 135° polarization direction, and *S*_3_ is the light intensity difference between left-handed and right-handed circularly polarized light. Generally, there is little circularly polarized light in the natural environment, so *S*_3_ is ignored in this article. By measuring the intensities of light along three or four different angles, the first three components of the Stokes parameters can be obtained. For example, measure the intensities of light along four directions of 0°, 45°, 90°, and 135°, which are respectively expressed by *I*_0_, *I*_45_, *I*_90_, and *I*_135_. The first three components of the Stokes parameters can be calculated as^29^:

1 $$\begin{aligned} S_{0} & = \frac{1}{2} \times (I_{0} + I_{45} + I_{90} + I_{135}) \\ S_{1} & = I_{0} - I_{90} \\ S_{2} & = I_{45} - I_{135} \\ \end{aligned}$$

By multiplying with Mueller matrix of a linear polarizor with axis at angle *θ,* the intensity image at any angle (denoted by *I*_*θ*_) can be derived from the first three components of the Stokes parameters as Eq. (2)^30^, in which the polarimetric images with the maximum and minimum variance can be selected.

2 $$I_{\theta} = \frac{1}{2} \times (S_{0} + S_{1}\cos 2\theta + S_{2}\sin 2\theta )$$

### Atmospheric scattering model and underwater scattering model

In a scattering environment, the total light captured by a camera *I*_*total*_ can be calculated from the sum of the attenuated object light *L·t* and the back scattered light *A*. Here, *L* is the clear object image and *t* is the transmittance of the scattering media. Generally, transmittance *t* is defined as Eq. (3), which relates the backscattered light *A* in the camera and the backscattered light from the infinite distance $A_{\infty }$.

3 $$t = 1 - \frac{A}{{A_{\infty } }}$$

4 $$L = \frac{I_{total} - A}{{1 - {A \mathord{\left/ {\vphantom {A {A_{\infty } }}} \right. \kern-\nulldelimiterspace} {A_{\infty } }}}} = \frac{{I_{total} - A_{\infty } (1 - t)}}{t}$$

So the restored image can be expressed by the Eq. (4)^35^. There are only two unknowns *t* and $A_{\infty }$, which need to be determined in the following restoration methods.

## Image restoration methods based on polarization imaging

**Figure 1:**
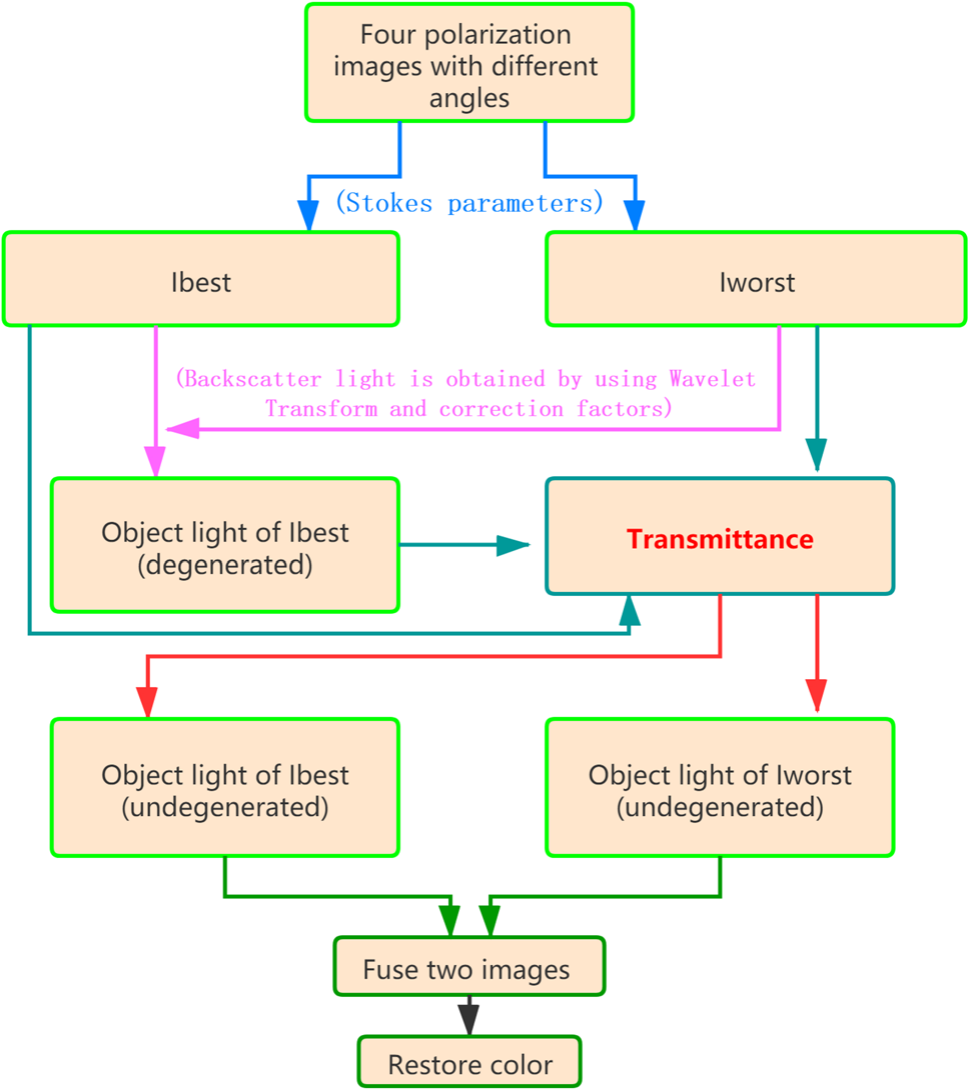
The process of the method proposed in this article.

In order to overcome the difficulty of polarization parameters estimation in a strong scattering scene with very weak polarized light, we adopted a restoration process based on the variance prior and illustrated it in Fig. 1. At first, the polarimetric images containing the most and least object light were selected among the calculation results of Eq. (2) with a variance prior, and named as *I*_*bes*t_ and *I*_*worst*_; then we calculated the backscattered light *A*_*w*_ from *I*_*worst*_ using a wavelet transform, and obtained the degenerated object light *D*_*b*_ from the difference between *I*_*best*_ and *A*_*w*_ multiplying with several polarimetric factors; furthermore, the transmittance of the media *t* was calculated by the combination of *D*_*b*_, *I*_*bes*t_, *I*_*worst*_ and the corresponding backscattered light; and the undegenerated object light in *I*_*best*_ was calculated as *L*_*b*_ = *D*_*b*_ / *t.* Similar process was used to calculate the undegenerated object light in *I*_*worst*_ and named a*s L*_*w*_*.* In the end, the sum of *L*_*b*_ and *L*_*w*_ was taken as the final restored image in our method. The detailed restoration algorithm was described as the following.

### Find the I_*best*_ and I_*worst*_

**Figure 2:**
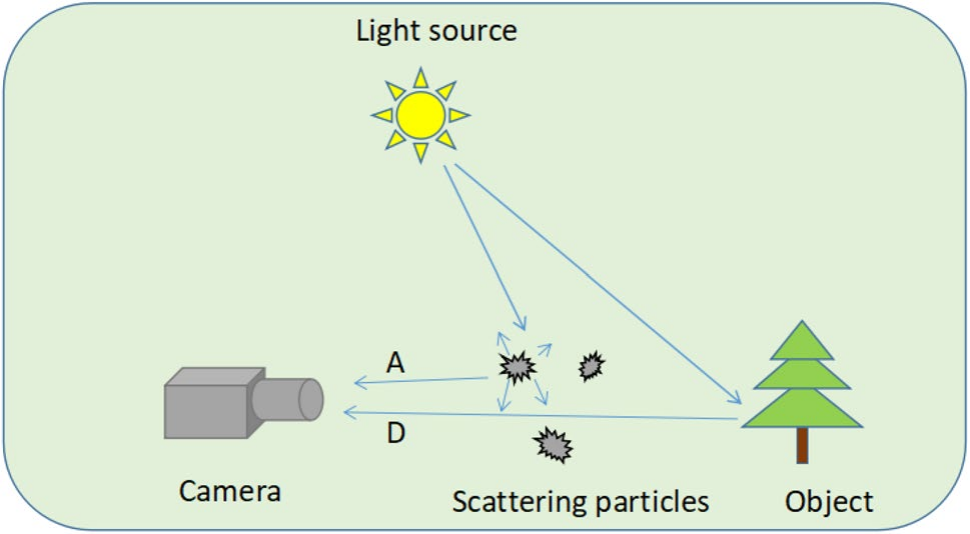
Imaging through a scattering medium.

The total light intensity received by the camera sensor is defined as *I*_*total*_, which is composed of the light from the object (defined as *D*) and the light from the background scattering medium (defined as *A*). This process is illustrated in Fig. 2.

In general, *D* and *A* have different polarization degrees and polarization angles. A linear polarizer is installed in front of an ordinary camera. Rotating the polarizer at different angles, the visibility of the image is different. At an angle *θ*, the light intensity *I*_*θ*_ can be written as

5 $$I_{\theta} = D_{\theta} + A_{\theta}$$

The angle at which the image contains the most object light can be defined as *θ*_*best*_, and the corresponding polarization intensity is defined as *I*_*best*_. The angle at which the image contains the least object light is defined as *θ*_*worst*_ and the corresponding intensity is *I*_*worst*_. At angle *θ*_*best*_, the value of *D*_*θ*_/*I*_*total*_ is maximum. At angle of *θ*_*worst*_, the value of *D*_*θ*_*/I*_*total*_ is minimum. In general, *θ*_*best*_ is perpendicular to *θ*_*worst*_. In previous work, *θ*_*best*_ and *θ*_*worst*_ are usually determined by the polarization property of a specific region. For example, using the brightest angle^8–10^, the direction perpendicular to the incident surface^11,22^, the polarization angle of the sky region^28^, or two reference objects^36^ to calculate *θ*_*best*_ were reported in former researches. In order to establish a universally applicable algorithm for various scattering media, we use a variance prior based on the fact that clear images usually have larger gray-scale variance and larger average gradient than images through scattering media^12^. The scattering medium overwhelms the details, reduce the contrast, gray-scale variance and average gradient^29^. Therefore, the gray-scale variance is taken as the standard to find *θ*_*best*_ and *θ*_*worst*_. Because the gray-scale variance does not depend on the polarization properties of a specific region of the image, it can be universally applied to various scattering media. In the specific calculation, the polarization image at any angle can be obtained by using Eq. (2). Each image at a different angle is divided into 10 × 10 blocks. The angle with maximum gray-scale variance in most regions is selected and regarded as *θ*_*best*_. *θ*_*worst*_ is perpendicular to *θ*_*best*_. After *θ*_*best*_ and *θ*_*worst*_ are selected, *I*_*best*_ and *I*_*worst*_ can be calculated by Eq. (2). The *θ*_*best*_ which maximizes the gray-scale variance of the image is automatically determined by the algorithm.

### Obtain the object light (degenerated) of I_*best*_

6 $$I_{best} = D_{b} + A_{b}$$

7 $$I_{worst} = D_{w} + A_{w}$$

Usually, the light intensity in a scattering media consists of object light *D* and backscattered light *A*. In Eqs. (6) and (7), *b* and *w* are the subscripts representing the images at *θ*_*best*_ and *θ*_*worst*_, respectively. After selecting the minimum variance region as background region, the polarization degree *P* can be derived from the background region of *I*_*best*_ and background region of *I*_*worst*_^9^.

8 $$P = \frac{I_{worst} - I_{best}}{{I_{worst} + I_{best}}} = \frac{A_{w} - A_{b}}{{A_{w} + A_{b}}}$$

From Eq. (8), we obtain:

9 $$A_{b} = A_{w} \times \frac{1 - P}{{1 + P}}$$

By combining Eqs. (6) and (9), we obtain:

10 $$D_{b} = I_{best} - A_{b} = I_{best} - A_{w} \times \frac{1 - P}{{1 + P}}$$

*A*_*w*_ and *P* are required to calculate *D*_*b*_. *A*_*w*_ is the backscattered light of *I*_*worst*_. Because background scattering media are relatively smooth compared with objects^12^, object light has a much higher frequency than the backscattered light. So the low-frequency component of *I*_*worst*_ (denoted by *A*′_*w*_) can be treated as an approximation of the backscattered light *A*_*w*_. Wavelet Transform is adopted to obtain the low-frequency component of *I*_*worst*_ in this article. For example, we use wavelet transform (basis function "db8") to take the fourth layer of low-frequency components of the image *I*_*worst*_ as *A*′_*w*_*.* There is still some object light that has not been removed in *A*′_*w*_. Two correction factors were introduced to estimate backscattered light more accurately.

#### First correction factor (denoted by *y*1)

For each channel, the correction factor component *y*_*i*_ (*i* = *r*, *g*, *b*) can be obtained by using the following formula:

11 $$i\;{\text{channel:}}\;\;y_{i} = \left| {\frac{{A^{\prime}w_{(i)} - A_{\infty }^{w} (i)}}{{A_{\infty }^{w} (i)}}} \right|$$

$A_{\infty }^{w}$ can be derived from the background region (average value of minimum variance region) of *I*_*worst*_, and note that $A_{\infty }^{w}$ is the backscattered light of *I*_*worst*_ from an infinite distance.

12 $$y1 = \frac{1}{{1 + \left( {y_{r} + y_{g} + y_{b}} \right)}}$$

The correction factor *y*1 is a matrix which is used to estimate the backscattered light of each pixel in *A′*_*w*_. It can be described simply as the reciprocal of the difference between a pixel and $A_{\infty }^{w}$. Less backscattered light in a pixel is corresponding to a smaller *y*1.

#### Second correction factor (denoted by *y*2)

When the polarized light is weak, the result of Eq. (10) is near zero. In order to avoid the unreasonable result, we introduce a correction factor *y*2 (also a matrix),

13 $$y2 = \left| {\frac{I_{worst} - I_{best}}{{I_{worst}}}} \right|$$

Using this two correction factors, Eq. (10) is rewritten as

14 $$D_{b} = I_{best} - A^{\prime}w \times y1 \times y2 \times \frac{1 - P}{{1 + P}}$$

In order to make our method applicable to more scenes and not only depend on the polarization degree *P*. We set *y* = (1 − *P*)/(1 + *P*), *P* is in the range [0–1]. Thus, *y* is in the same range of [0–1]. In the actual algorithm, the image with maximum average gradient was calculated by searching the exact *y* in the range of [0–1]. The parameter *y* which maximizes the average gradient of the image is automatically determined by the algorithm.

In this section, the wavelet transform and two correction factors were used to calculate *D*_*b*_.

### Calculate the transmittance

Because *I*_*best*_ and *I*_*worst*_ are two images of the same scene at two angles which are perpendicular to each other. We assume that they have the equivalent transmittance *t*. According to Eq. (4),

15 $$t = 1 - \frac{A_{b}}{{A_{\infty }^{b} }} = 1 - \frac{A_{w}}{{A_{\infty }^{w} }}$$

where $A_{\infty }^{b}$ and $A_{\infty }^{w}$ can be respectively derived from the background region (average value of minimum variance region) of *I*_*best*_ and *I*_*worst*_, and *A*_*w*_ is unknown. *t* can be calculated by *A*_*b*_ and $A_{\infty }^{b}$. However, *I*_*best*_ contains most of the object light and a small part of scattered light, *I*_*worst*_ contains a small part of the object light and most of the scattered light. $A_{\infty }^{b}$ is little and as a denominator it causes the instability of the result. As an alternative, *t* is calculated by Eq. (17).

16 $$\frac{A_{b}}{{A_{\infty }^{b} }} = \frac{A_{w}}{{A_{\infty }^{w} }} = C$$

17 $$t = 1 - C = 1 - \frac{A_{b} + A_{w}}{{A_{\infty }^{b} + A_{\infty }^{w} }} = 1 - \frac{I_{best} + I_{worst} - D_{b} - D_{w}}{{A_{\infty }^{b} + A_{\infty }^{w} }} \approx 1 - \frac{I_{best} + I_{worst} - D_{b}}{{A_{\infty }^{b} + A_{\infty }^{w} }}$$

As mentioned above, *D*_*w*_ is much smaller than *D*_*b*_. It can be neglected in Eq. (17). So after calculation of *I*_*best*_ and *I*_*wors*_ from the captured polarization images, $A_{\infty }^{b}$ and $A_{\infty }^{w}$ from the background region, *D*_*b*_ from Eq. (14), the transmittance of the scattering media can be computed.

### Obtain the object light (undegenerated) and fuse two images

Now we can obtain the object light *L*_*b*_ of the image *I*_*best*_, which has not been degraded.

18 $$L_{b} = \frac{D_{b}}{t}$$

And the object light *L*_*w*_ of the image *I*_*worst*_ can be derived from Eq. (3):

19 $$L_{w} = \frac{{I_{worst} - A_{\infty }^{w} (1 - t)}}{t}$$

The fused object light *L* is obtained:

20 $$L = L_{b} + L_{w}$$

We have used transmittance *t* to obtain object light *L*. In the last step of our method, the normalized first component of Stokes parameters *S*_0_ is used for color restoration. *L* was multiplied by the gray value of *S*_0_ to correct the intensity of each color channel.

## Experimental results and discussion

**Figure 3:**
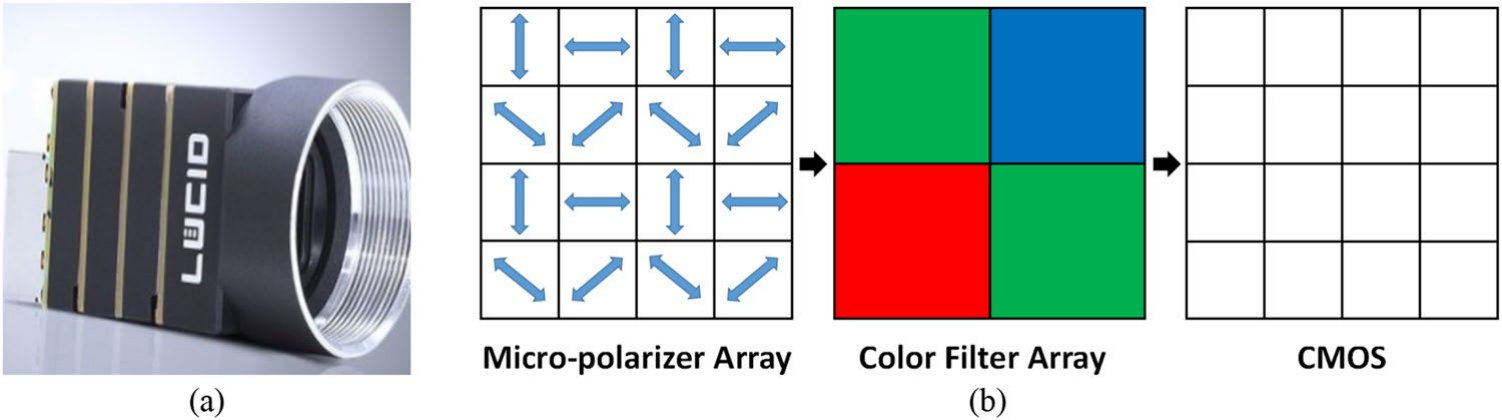
(**a**) is polarization camera, (**b**) is the smallest periodical cell in camera focal plane.

**Figure 4:**
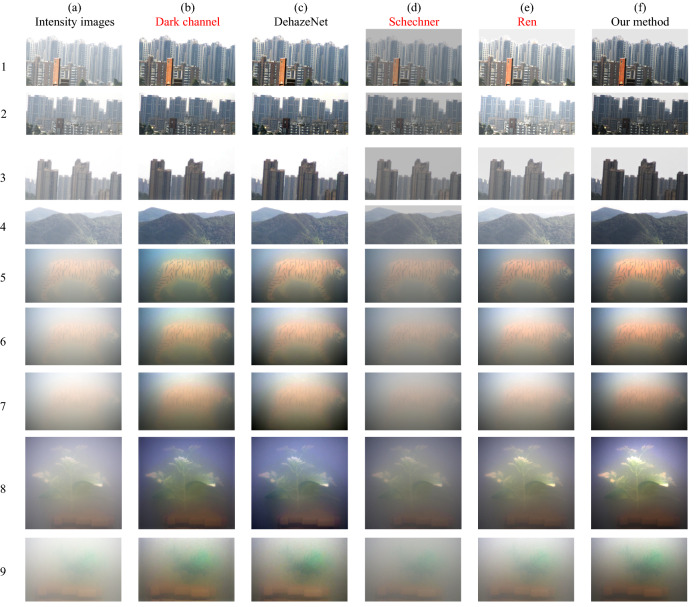
The groups of 1–4 are the buildings and hills in the foggy weather. The groups of 5–9 are the underwater experiments in the milk solution with different concentrations. (**a**) is intensity image (*S*_0_), (**b**) is the restored image with dark channel prior, (**c**) is the restored image with DehazeNet, (**d**) is the restored image with Schechner’s method, (**e**) is the restored image with Ren’s method, (**f**) is the restored image with proposed method.

We used a polarization camera (Luccid PHX050S, Canada) based on the technology of division of the focal plane, which was capable to capture four polarization images with different angles (0°, 45°, 90°, 135°) at the same time and can be applied to a moving object. The polarization camera is shown in Fig. 3(a). The smallest periodical cell in the camera focal plane is shown in Fig. 3(b). Micro-polarizer array and color filter array are in front of the sensor. The underwater experiments were arranged in a square glass container, and the background behind the glass container was arranged in black. The light source (LED white light source) was used for illumination. The intensity images (*S*_0_), the results of dark channel prior dehazing, the results of DehazeNet^6^, the results of Schechner’s method, the results of Ren’s method^34^ and the results of our proposed method are given in Fig. 4. The groups of 1–4 are images of outdoor buildings and hills in the foggy weather, the distance between the scene and the camera is within a few thousand meters. And the groups of 5–9 are indoor underwater experiments in the milk solution with different concentrations. The imaging objects were put in a transparent water tank made of glasses, and A 5 W unpolarized white LED light was taken as the illumination source in front of the tank.

In Fig. 4, in group 1–4, the objects are buildings and hills in the foggy weather. (a) is the intensity image, (b)-(f) are the corresponding restored images using dark channel prior, DehazeNet, Schechner’s method, Ren’s method, and our method, respectively.

**Figure 5:**
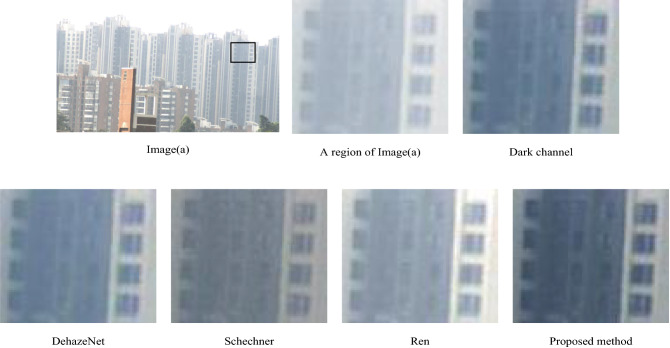
The same region in images (**a**), (**b**), (**c**), (**d**), (**e**), (**f**) in group 1.

**Figure 6:**
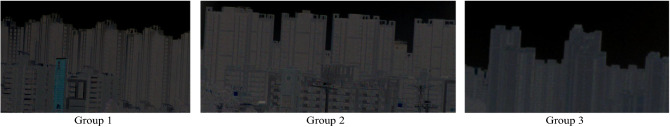
Images of polarization degree in group 1–3.

As we can see, in group 1, the building in intensity image (a) is very blurry. Buildings in images (b), (c) and (f) are clearer. For more details, we choose the same region (black frame) in images (a-f) and enlarge it in Fig. 5. Obviously, the proposed method obtains the clearest restored image with most details. It causes less noise and restored more details in the image. Same results can be found in group (1–3), even the buildings in group 3 are much farther away from the observer than those in group 1 and 2. Long distance scattering in fog causes the depolarization of the light, which results in the restoration difficulty in a method using polarization prior. For comparison, images of polarization degree (*P* = (*S*_1_^2^ + *S*_2_^2^)^0.5^/*S*_0_) are displayed in Fig. 6. As showed in Fig. 6, the object light is polarimetric and air light is not polarimetric. So Schechner’s polarization assumption is not accurate here. Images (d) in group 1–3 in Fig. 4 show Schechner’s method does not work very well. Our method does not depend on the specific state of polarization and is more universal. In group 4, hills are shrouded in fog. The color in the image (f) is closest to the real scene. It is dark green and little fog on the hill in front of the scene.

The concentration of the milk powder increases gradually from group 5 to 7. and from 8 to 9. In group 5 (10 mL milk in 6L water), 6(12.5 mL milk in 6L water) and 7(15 mL milk in 6L water), from images (c) and (f), we found the results of DehazeNet and proposed method are much better than intensity image (*S*_0_). And severe blue shift happens in image (b), while the true color of the image is kept in our method. By comparison of restored images with different concentration of milk, we found that Schechner’s method is better than the image (*S*_0_) but not very effective with little polarimetric light. Especially in high-concentration scattering media, the degree of polarization is very small. But the proposed method still works well under such strong scattering condition.

**Figure 7:**
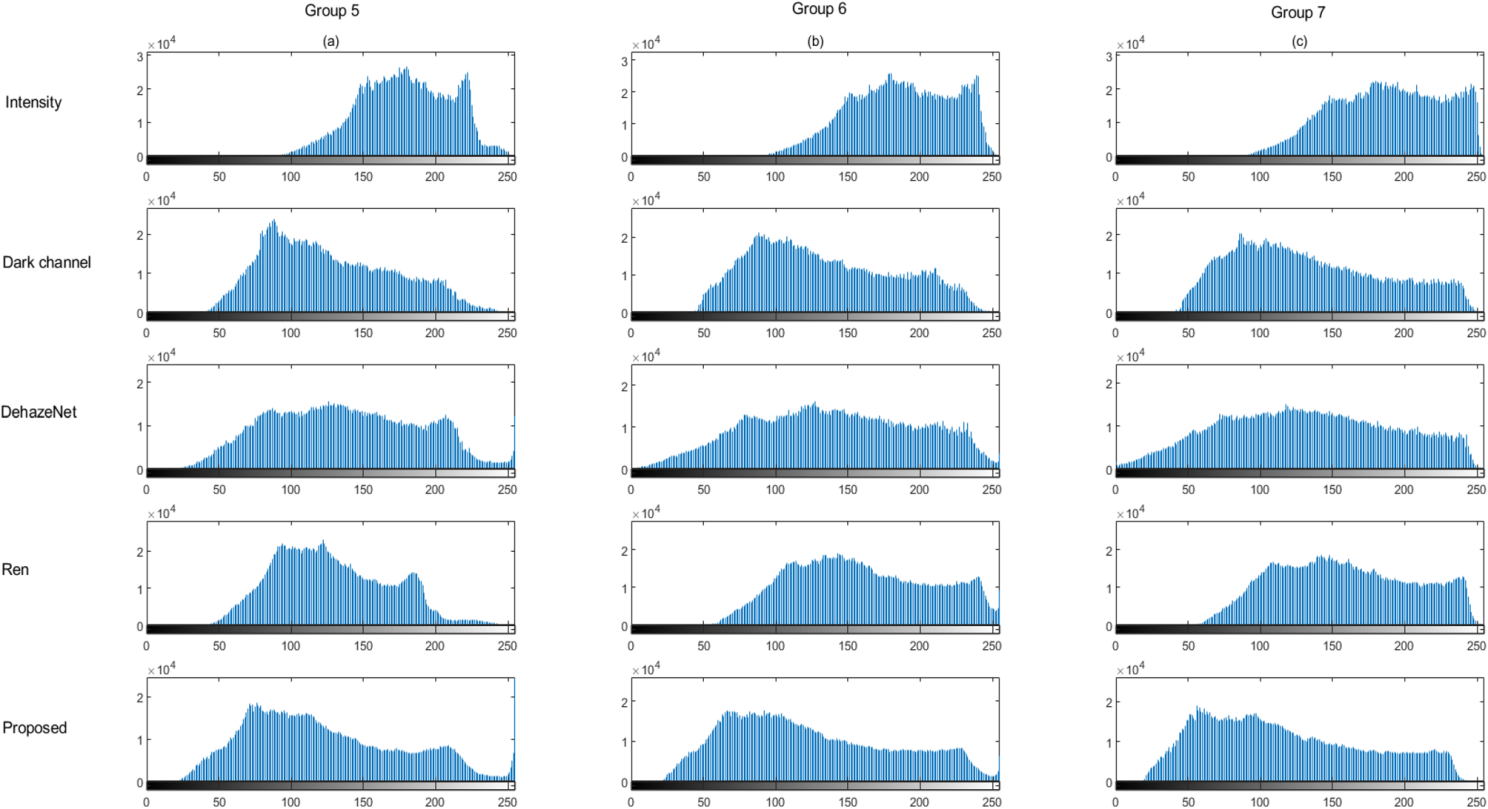
x-axis is frequency, y-axis is gray level. (**a**), (**b**) and (**c**) are the histograms of images in Group 5–7 in Fig. 4, respectively. The intensity images and the corresponding restored images are listed indifferent lines.

The histograms can provide a more accurate description on the spectrum and details in an image. We calculate the histograms of the images in the group 5–7 in Fig. 4 and plotted in Fig. 7. Wider histogram relates to an image with higher quality and more details. We find that the histograms of both DehazeNet and our proposed method in Group 5–7 are wider than others in Fig. 7.

**Figure 8:**
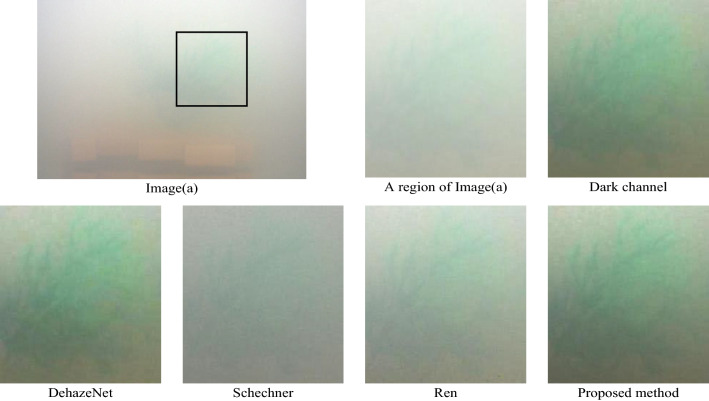
The same region in images (**a**), (**d**), (**e**), (**f**) in group 9.

In order to observe the details, we choose the same region (black frame) and enlarge them in Fig. 8. In such a strong scattering, the intensity image (a) in group 9 is very blurry and we cannot see any details in this image. The restored images with Schechner’s method and with Ren’s method are better than the intensity image. But it is still blurry. The restored images with dark channel method, with DehazeNet and with the proposed method are much better.

Next, we evaluate the experimental results by four objective evaluation indexes: contrast^1^, gray standard deviation^37^, average gradient^38^ and information entropy^7^. Contrast reflects the difference between adjacent pixels in the image. The better image has larger contrast. Gray standard deviation reflects the degree that the gray value deviates from the average value. The better image has larger standard deviation. Average gradient reflects the gray value change rate of the image. The larger average gradient of image is, the better details of the image are. Information entropy reflects the amount of information contained in an image. An image with larger information entropy contains more information.

In Table 1, the bold data indicates the maximum value of every group, and the percentage in brackets indicates the improvement of the proposed method compared with the intensity image. As can be seen from Table 1, compared with the intensity image, contrast, gray standard deviation, average gradient and information entropy of the restored images are improved evidently. In general, compared with intensity images, the standard deviation and average gradient of restored images by the proposed method are enhanced by 50–100%. The information entropy is improved by 5–10%. And the contrast is enhanced by 100–300%.

**Table 1:** Objective evaluation indexes of images.

Group	Image	Contrast	Gray standard deviation	Average gradient	Information entropy
1	Intensity	4.444	15.362	10.965	6.156
	Dark channel	10.96	24.385	17.046	6.539
	DehazeNet	**13.051**	**26.096**	**18.483**	6.69
	Schechner	4.615	13.174	11.115	6.04
	Ren	12.967	19.832	17.54	6.6
	Proposed	12.099 (172.3%)	25.482 (65.9%)	17.48 (59.4%)	**6.752** **(9.7%)**
2	Intensity	4.132	15.91	10.864	6.589
	Dark channel	7.94	23.423	14.396	6.839
	DehazeNet	**10.164**	**25.114**	**16.385**	**7.13**
	Schechner	4.644	14.053	11.283	6.41
	Ren	8.75	17.208	15.134	6.461
	Proposed	8.043 (94.6%)	24.735 (55.5%)	13.739 (26.5%)	6.854 (4.0%)
3	Intensity	0.845	17.628	4.378	5.162
	Dark channel	1.956	29.818	5.873	5.393
	DehazeNet	**2.436**	**31.616**	**6.946**	5.619
	Schechner	0.767	15.254	4.261	4.826
	Ren	1.279	19.822	5.135	5.396
	Proposed	2.362 (179.5%)	31.206 (77.0%)	6.522 (49.0%)	**5.652** **(9.5%)**
4	Intensity	0.622	13.717	4.868	6.004
	Dark channel	1.318	22.027	6.482	6.126
	DehazeNet	1.287	21.007	6.572	6.019
	Schechner	0.727	12.69	5.191	5.921
	Ren	1.037	16.94	6.337	6.378
	Proposed	**1.444** **(132.3%)**	**25.668** **(87.1%)**	6.486 (33.2%)	**6.544** **(9.0%)**
5	Intensity	0.123	11.985	1.869	6.862
	Dark channel	0.348	17.052	3.204	7.308
	DehazeNet	0.379	19.529	3.467	**7.535**
	Schechner	0.254	10.031	2.89	6.615
	Ren	0.42	14.997	**3.748**	7.179
	Proposed	**0.446** **(263.9%)**	**20.714** **(72.8%)**	3.678 (96.8%)	7.531 (9.7%)
6	Intensity	0.081	13.398	1.522	7.003
	Dark channel	0.223	18.811	2.625	7.458
	DehazeNet	0.259	**21.984**	2.961	**7.747**
	Schechner	0.179	11.166	2.43	6.755
	Ren	**0.372**	18.537	**3.624**	7.465
	Proposed	0.277 (239.9%)	21.899 (63.5%)	3.065 (101.4%)	7.658 (9.4%)
7	Intensity	0.07	14.488	1.43	7.108
	Dark channel	0.188	19.95	2.409	7.526
	DehazeNet	0.233	**22.701**	2.838	**7.796**
	Schechner	0.146	11.998	2.183	6.853
	Ren	**0.246**	18.112	**2.869**	7.439
	Proposed	0.207 (194.3%)	21.707 (49.8%)	2.638 (84.5%)	7.638 (7.5%)
8	Intensity	0.073	11.228	1.368	6.737
	Dark channel	0.164	12.298	2.057	6.823
	DehazeNet	0.199	15.455	2.412	7.168
	Schechner	0.212	9.485	2.605	6.522
	Ren	0.21	13.593	2.474	7.021
	Proposed	**0.298** **(307.0%)**	**20.046** **(78.5%)**	**2.946** **(115.3%)**	**7.518** **(11.6%)**
9	Intensity	0.073	11.238	1.414	6.667
	Dark channel	0.207	15.315	2.47	7.106
	DehazeNet	**0.244**	15.695	**2.856**	7.188
	Schechner	0.162	9.623	2.254	6.462
	Ren	0.231	15.34	2.793	7.116
	Proposed	0.222 (203.5%)	**19.026** **(69.3%)**	2.775 (96.3%)	**7.381** **(10.7%)**

The bold data indicates the maximum value of every group.

So far, our defogging algorithm has a performance as good as DehazeNet, both in foggy weather and underwater experiments. DehazeNet is a latest-developed dehazing method using a convolutional neural network^6^. It has an extremely high restoration accuracy, but requires a network training based on a large number of pre-collected image libraries. Our method can directly defog in a single image, so it will be more practical in the application of various scattering environment.

## Conclusion

In this article, we proposed an image restoration method based on gray variance and average gradient assumption to overcome the difficulty of polarization parameter estimation under strong scattering condition. This technology is performed with a single-shot polarization camera based on the technology of division of the focal plane. Experimental results show that the proposed method obtains clear restored images. Same improvements can be found in the evaluation indexes for our method. Therefore, compared with previously reported methods, our method has some advantages of more restoration details, less noise, and more general applicability. It is independent of a specific polarization state in various scenes and keeps effective even in a scene only containing little polarized light.
